# Vitamin D may alleviate irritable bowel syndrome by modulating serotonin synthesis: a hypothesis based on recent literature

**DOI:** 10.3389/fphys.2023.1152958

**Published:** 2023-07-27

**Authors:** Xiao-Lan Yu, Cui-Ping Li, Lian-Ping He

**Affiliations:** School of Medicine, Taizhou University, Jiaojiang, Zhejiang, China

**Keywords:** depression, irritable bowel syndrome, vitamin D, serotonin, hypothesis, literature

## Abstract

A number of studies found that serotonin plays a vital role in the development of depression and irritable bowel syndrome. Recent studies showed that vitamin D was associated with regulating the synthesis of serotonin. This review focuses on the recent progress in the relationship between vitamin D and serotonin synthesis.

## Introduction

**FIGURE 1:**
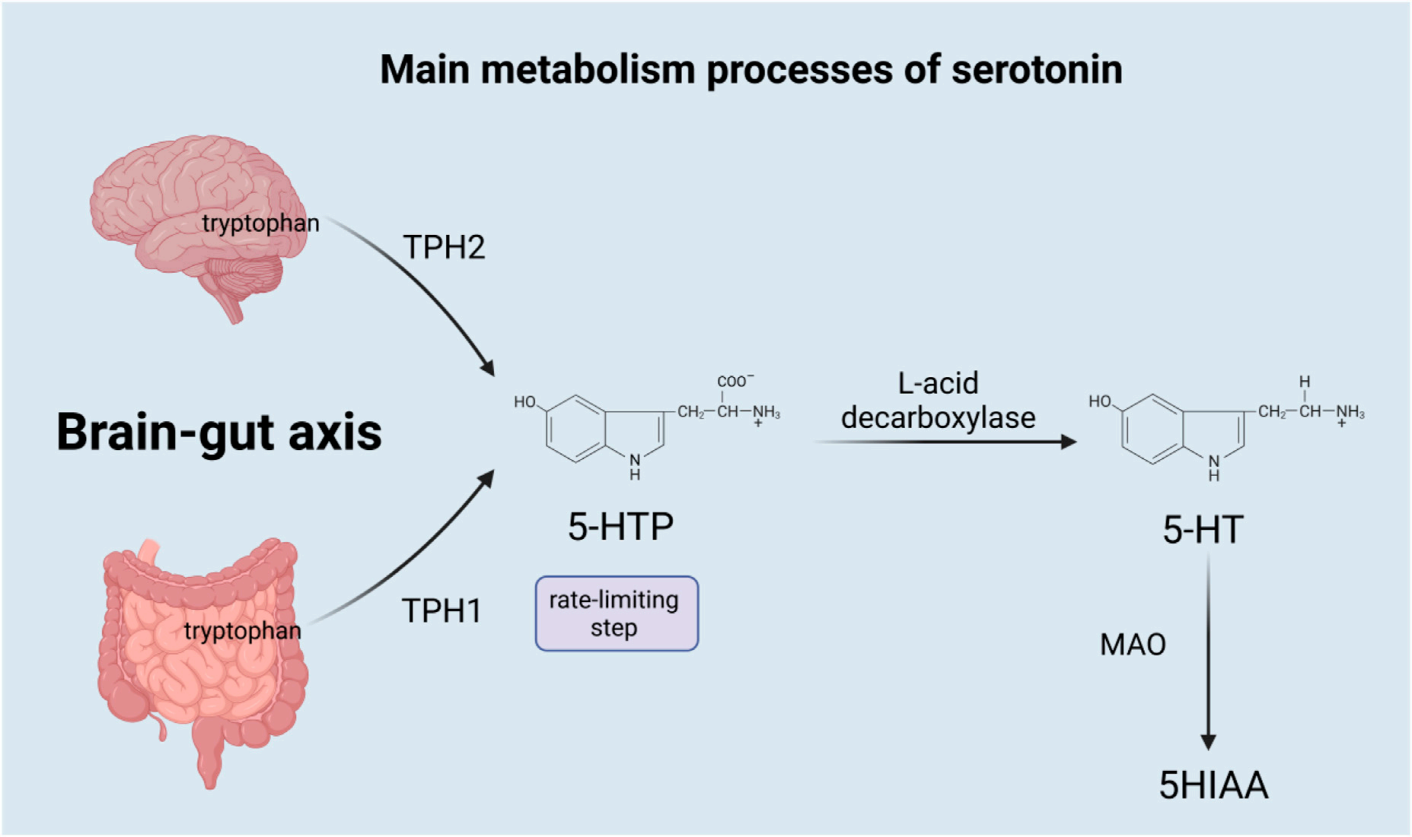
TPH1, tryptophan hydroxylase 1; TPH2, tryptophan hydroxylase 2; 5-HTP, 5-hydroxytryptophan; 5-HT, 5-hydroxytryptamine; MAO, monoamine oxidase; and 5HIAA, 5-hydroxyyin polyacetate.

Serotonin is a monoamine neurotransmitter derived from tryptophan (Mathur and Lovinger, 2012), synthesized both centrally and systemically, and released throughout the brain. The synthesis of serotonin is mainly divided into two steps: the first step is a rate-limiting step, where tryptophan is hydroxylated to 5-hydroxytryptophan (5-HTP) by tryptophan hydroxylase (TPH). 5-HTP is subsequently decarboxylated by aromatic L-acid decarboxylase to eventually form 5-hydroxytryptamine (5-HT) (Sahu et al., 2018), which is also called serotonin. However, most of the serotonin is synthesized in the gastrointestinal tract, while only a small amount is produced in the nervous system (Charnay and Leger, 2010). Due to different genes, TPH is divided into TPH1 and TPH2, among which TPH1 mainly exists in non-brain tissues, such as the gut enterochromaffin cells, pineal gland, placenta, and T cells (Leon-Ponte et al., 2007), and is responsible for producing most of the serotonin in the body; TPH2 exists in the neurons of the raphe nuclei and the enteric nervous system, responsible for the production of serotonin in the brain (Gutknecht et al., 2009), which acts as a neurotransmitter in the central nervous system (CNS) (Sahu et al., 2018). Serotonin has two main sources in the gut: the enterochromaffin (EC) cells of the mucosa contain the vast majority of serotonin, and myenteric neurons that project in descending pathways contain a little (Kendig and Grider, 2015). Almost all serotonin in the blood is located on platelets, which absorb serotonin from the gut and transport it to the blood (Chen et al., 2001). In the brain, gastrointestinal tract, lung, liver, and platelets, serotonin is mainly metabolized by monoamine oxidase (MAO) to 5-hydroxyyin polyacetate (5HIAA), which is excreted through urine. Serotonin is also metabolized through the glucuronidation and sulfation pathways, which occur in the liver, lung, kidney, and brain (Mohammad-Zadeh et al., 2008). Figure 1 summarizes the main metabolism processes of serotonin.

### Effect of vitamin D on irritable bowel syndrome

Vitamin D (VD) is a fat-soluble steroid that is a key regulator of calcium and phosphorus metabolism (Holick, 2007), which mainly circulates to form 25-hydroxyvitamin D3 [25 (OH)D3]. Recently, studies have found that VD plays a vital role in development of depression (Kaviani et al., 2020), irritable bowel syndrome (IBS) (Alvi et al., 2022), type 1 diabetes mellitus (Liu et al., 2015), and obesity (Yu et al., 2023; He et al., 2023; Li et al., 2023). Current research suggests that VD can interfere with a plethora of cellular mechanisms and directly or indirectly modulate the microbiota (Tangestani et al., 2021), release antimicrobial peptides, and modulate the immune system and inflammatory processes (Song et al., 2023) this, in turn, can actively interfere with changes in gut function.

IBS is a common chronic functional gastrointestinal disorder characterized by abdominal pain, bloating, urinary urgency, voiding incontinence, and altered bowel habits associated with structural and biochemical abnormalities (Altomare et al., 2021). The mechanism of its occurrence is not yet clear, and current research shows that it may be related to the gut–brain axis, serotonin pathway, and gut microbiota (Bonetto et al., 2021). A number of studies have shown that IBS patients are often accompanied by lack of VD (Khayyat and Attar, 2015; Nwosu et al., 2017; Cho et al., 2018; Chong et al., 2022), intestinal microbiota disturbance (Pittayanon et al., 2019), immune dysregulation (Ng et al., 2018), anxiety, and depression (Bercik, 2020). VD increases the relative abundance of beneficial bacteria and reduces the microbial composition of *Firmicutes* (Huang et al., 2022), which significantly improves the intestinal microbiota, and it also acts as an immunomodulator, increasing the production of antimicrobial peptides, regulating the integrity of intestinal epithelial cells, and inhibiting Th1/Th17 cells, while alleviating Treg cells to inhibit intestinal inflammation (Lopez et al., 2021), so as to improve IBS.

### Serotonin was associated with irritable bowel syndrome

It is currently believed that serotonin is related to various mental diseases such as depression, while regulating peripheral intestinal function (Jones et al., 2020).

Through rectal biopsies from healthy controls and patients with ulcerative colitis (UC), IBS with diarrhea (IBS-D), and IBS with constipation (IBS-C), it was found that colonic mucosal serotonin concentrations in UC, IBS-C, and IBS-D specimens were significantly lower than those in healthy control specimens (Coates et al., 2004). However, interestingly, in another experiment, serotonin in the rectal mucosa of IBS-D patients was higher than normal after a meal (Kumar et al., 2012), which may worsen the symptoms (Houghton et al., 2003). The resident microbiota has direct and indirect regulation of tryptophan and serotonin in the gut. Another experiment showed that gut microbiota can promote serotonin in colonic endothelial cells in an inducible and reversible manner, playing a key role in increasing colonic and serum 5-HT levels (Yano et al., 2015). At the same time, it can also control the central serotonergic system (SS) by regulating peripheral serotonin (Clarke et al., 2013). In addition, the brain–gut axis is a two-way communication system between the brain and the gastrointestinal (GI) tract, linking the emotional and cognitive centers of the brain to the peripheral control and function of the gut (Jenkins et al., 2016). However, it needs to be emphasized that the brain and gut serotonin systems are separated by the blood–brain barrier (BBB), and serotonin in the gut cannot penetrate the BBB (Bektas et al., 2020; Yaghoubfar et al., 2020).

### VD in the regulation of serotonin synthesis

Serotonin regulates a wide variety of brain functions and behaviors. Previous findings have demonstrated that serotonin regulates executive function, sensory gating, and social behavior and that attention deficit hyperactivity disorder, bipolar disorder, schizophrenia, and impulsive behavior all share in common defects in these functions. Serotonin synthesis was controlled by VD (Patrick and Ames, 2014; Patrick and Ames, 2015). There is also a clinical association of VD and serotonin levels among patients with fibromyalgia syndrome (Amin et al., 2019).

Nowadays, distinct vitamin D response elements (VDREs) have been identified in the regulatory regions of both TPH2 and TPH1 (Patrick and Ames, 2015), which suggests that VD may affect the regulation of central and blood serotonin concentrations through the transcriptional regulation of TPH gene expression (Wang et al., 2020). The experimental data of the work of Ichiro K et al. also showed that VD can control the synthesis of serotonin in related areas of the brain, which may be due to the enhancement of TPH2 in the brain (Kaneko et al., 2015). Regarding serotonin, VD not only upregulates the transcription of the serotonin synthesis gene TPH2 in the brain but also represses TPH1 in other tissues to maintain normal serotonin levels in the body (Patrick and Ames, 2014). Serotonin in the gut has been shown to be a very strong proliferation signal for T cells, and inhibition of TPH1 and serotonin production in the gut can improve related inflammatory symptoms (Gustafson, 2014).

In people with insufficient VD levels, VD supplementation may improve the mood of patients by increasing the availability of serotonin in the brain through the expression of TPH2 (Huiberts and Smolders, 2021), while in IBS patients, the disturbance of gut microbiota affects serotonin synthesis in intestinal chromaffin cells (Linsalata et al., 2023), which leads to chronic abdominal pain, and VD can indirectly affect serotonin by improving gut microbiota to relieve abdominal pain.

These findings provide important evidence that VD affects the synthesis of serotonin to relieve anxiety and inflammation symptoms in patients, thereby improving IBS.

### Experiments and studies of VD and serotonin

Marya SS et al. quantified the mRNA expression of serotonin synthesis isoenzymes TPH1 and TPH2 as well as the serotonin reuptake transporter (SERT) and the enzyme responsible for serotonin catabolism monoamine oxidase-A (MAO-A) expression to explore the response of neuronal cells to 1.25D. The results show that 1.25D not only induces serotonin synthesis but also acts at an indirect molecular genomic stage, mimicking SSRI and MAO inhibitors, which may elevate serotonin in the central nervous system, and this also explains the effect of VD on neuronal trajectories and causes of development of mental disorders (Sabir et al., 2018). Meanwhile, another study also reported that a low-VD diet reduced the brain serotonin concentration in mature female mice, providing evidence for the link between VD and serotonin (Wang et al., 2020).

Although VD has TPH2 expression in cultured rat serotonergic neurons, a randomized clinical trial of VD supplementation in children with autism spectrum disorder (ASD) showed that VD supplementation had no significant effect on serum serotonin levels of ASD children (Javadfar et al., 2020). Additionally, there is currently a lack of clinical trials on the effect of VD supplementation on serum serotonin, which is a direction for future development and requires further research. In a word, research on VD and serotonergic pathways is of great significance not only for the treatment of mental disorders but also for the mental health of healthy people (Huiberts and Smolders, 2021).

## Future direction

Numerous studies have shown that serotonin is related to mental and intestinal functions, and VD may affect serotonin levels by regulating TPH, thereby affecting human mental and intestinal functions. This has been confirmed in animal experiments, but there is a lack of sufficient evidence in clinical trials, which provides directions for future research.
